# The effects of gonadotropin-releasing hormone agonist on final adult height among girls with early and fast puberty

**DOI:** 10.3389/fendo.2023.1271395

**Published:** 2023-11-03

**Authors:** Chin-Hui Tseng, Yann-Jinn Lee, Chi-Yu Huang, Yi-Lei Wu, Lu-Ting Wang, Chao-Hsu Lin, Bi-Wen Cheng, Fu-Sung Lo, Yu-Jun Chang, Wei-Hsin Ting

**Affiliations:** ^1^ Department of Pediatric Endocrinology, MacKay Children’s Hospital, Taipei, Taiwan; ^2^ Department of Pediatric Endocrinology and Metabolism, Chuanghua Christian Childrens Hospital, Changhua, Taiwan; ^3^ Department of Medicine, MacKay Medical College, New Taipei City, Taiwan; ^4^ Department of Medical Research, Tamsui MacKay Memorial Hospital, New Taipei City, Taiwan; ^5^ Institute of Biomedical Sciences, MacKay Medical College, New Taipei City, Taiwan; ^6^ Department of Pediatrics, School of Medicine, College of Medicine, Taipei Medical University, Taipei, Taiwan; ^7^ Department of Pediatric Endocrinology, Hsinchu MacKay Memorial Hospital, Hsinchu, Taiwan; ^8^ Department of Biological Science and Technology, National Yang Ming Chiao Tung University, Hsinchu, Taiwan; ^9^ Department of Pediatric Endocrinology, Chang Gung Memorial Hospital, Taoyuan, Taiwan; ^10^ College of Medicine, Chang Gung University, Taoyuan, Taiwan; ^11^ Big Data Center, Changhua Christian Hospital, Changhua, Taiwan

**Keywords:** GnRH agonist, early and fast puberty, final adult height, central precocious puberty, girls

## Abstract

**Introduction:**

This study aimed to explore the impact of gonadotropin-releasing hormone agonists (GnRHa) on final adult height (FAH) in girls with early and fast puberty.

**Methods:**

A retrospective study was conducted by reviewing data from the medical records of the Pediatric Endocrinology Clinics between January 1, 2010, and December 31, 2020, at MacKay Children’s Hospital. The treatment group included 109 patients who received 3.75 mg monthly for at least 1 year, whereas the control group consisted of 95 girls who received no treatment.

**Results:**

The treatment group was significantly older at the time of inclusion(chronological age (CA1), treatment vs. control, 8.7 vs. 8.4 years, p < 0.001), had a more advanced bone age (BA) (BA1, 11.5 vs. 10.8 years, p < 0.001), BA1-CA1 (2.7 vs. 2.2 years, p < 0.001), and shorter predicted adult height (PAH1) (153.3 vs. 157.1 cm, p = 0.005) that was significantly lower than their target height (Tht)(PAH1-Tht, −3.9 vs. −1.3 cm, p = 0.039). The FAHs of the GnRHa and the control group were similar (157.0 vs. 156.7 cm, p = 0.357) and were not significantly different from their Tht (FAH vs. Tht in the GnRHa group, 157.0 vs. 157.0 cm; control group, 156.7 vs. 157.0 cm). In the subgroup analysis, FAH was significantly higher after GnRHa treatment in those with PAH1 less than 153 cm and Tht (154.0 vs. 152.0 cm, p = 0.041), and those whose CA1 was between 8 and 9 years (158.0 vs. 155.4 cm, p = 0.004). We defined satisfactory FAH outcome as FAH-PAH1≥5 cm and significant factors were GnRHa therapy, PAH1 shorter than their Tht, age younger than 9 years, and faster growth velocity during the first year.

**Discussion:**

GnRHa is effective in restoring the Tht in some early and fast pubertal girls, especially in those with poorly PAH (PAH lower than 153 cm and shorter than their target height). A younger age at initiation of treatment and a faster growth velocity during treatment are associated with a better height gain.

## 1 Introduction

Precocious puberty is defined as the onset of the physical signs of puberty before the age of 8 years in girls and 9 years in boys, leading to the progressive development of secondary sexual characteristics and bone maturation. Central precocious puberty (CPP) is the result of premature reactivation of the hypothalamic–pituitary–gonadal (HPG) axis and pulsatile GnRH secretion and is associated with diminished height potential due to early maturation of the growth plate (1, 2). If puberty occurs within the first half of the normal distribution of pubertal onset, it is defined as early puberty (EP) (3, 4). Because pubertal timing is highly variable, various age intervals have been mentioned in the literature for EP in girls, such as between 8 and 10 years in Europe (3) and 7–9 years in the United States (5). Some patients with EP present with rapid pubertal development, such as reaching Tanner stage 3 before 10 years of age (6, 7), which is often associated with bone age advancement, raising concerns about compromised final adult height (FAH) in this group.

Gonadotropin-releasing hormone agonists (GnRHa) were introduced more than 30 years ago and have become the treatment of choice for children with CPP, as these have been shown to restore genetic growth potential (4, 8–12). Consensus regarding the use of GnRHa analogs in 2009 showed that GnRHa therapy increases FAH in girls with CPP who begin puberty before the age of 6 but the effect on girls whose puberty occurs at an older age is controversial (13). Two randomized controlled trials with a limited sample size conducted in the 1990s revealed no obvious effect of GnRHa on improving FAH in patients with pubertal onset between the ages of 7.5 and 10 years (4, 14). Owing to parental anxiety about the effects of early and fast puberty on FAH, randomized controlled trials are unlikely to be conducted. However, FAH outcomes in patients with early and fast puberty remain to be investigated. Retrospective cohort studies with a control group demonstrated an inadequate effect of GnRHa in improving FAH. Lazar et al. (15) analyzed the data of 126 girls whose puberty started at 8–9 years (63 treated with GnRHa and 63 untreated). The treated and untreated girls achieved a similar final height, and they concluded that GnRHa affected only the pace of EP but not the total pubertal growth or final height. Savaş-Erdeve et al. analyzed fewer cases aged 7–8.5 years with EP and divided the treated group into two groups according to the degree of bone age advancement. The final heights were similar between groups. Observational studies, such as those by Ibanez et al. (16), revealed that girls with normal birth weight and pubertal age between 8 and 9 years tended to progress slowly through puberty, with a normal final height. Jaruratanasirikul et al. (17) studied the pattern of pubertal development in Thailand in up to 104 girls whose onset of puberty between 7 and 9 years successfully achieved target height (Tht) without treatment.

However, other prospective (8) and retrospective studies (11, 18–22) have shown that GnRHa is effective in improving height outcomes in girls with EP. Chiavaroli et al. (8) suggest that GnRHa therapy is helpful in reaching an adult stature close to the Tht in girls with a median pubertal age of 8.9 years. In 2020, Fu et al. (22) recruited 448 Chinese girls, 276 of whom were treated with the GnRHa. They showed that the difference between FAH and predicted adult height (PAH) was significantly different between the GnRHa and rhGH treatment, GnRHa alone, and no-treatment groups. The overall beneficial effects of the GnRHa on FAH were significant. Systematic reviews attempting to determine this have been conducted but they drew different conclusions (6, 7, 23, 24).

The inconsistency in results across studies has led to some degree of confusion and uncertainty in making treatment recommendations for girls with early and fast puberty, who represent a large proportion of patients seeking medical advice. Therefore, the objective of our study was to examine the effects of the GnRHa on FAH in girls with early and fast puberty. We also explored the possible factors related to FAH in girls with early and fast puberty with and without treatment.

## 2 Materials and methods

### 2.1 Participants

A retrospective study was conducted by reviewing data from a single medical center. Data were collected from the medical records of the Pediatric Endocrinology Clinics between 1 January 2010 and 31 December 2020 at MacKay Children’s Hospital, a tertiary medical center and medical research institution in northern Taiwan.

The inclusion criteria were as follows: (1) girls with breast Tanner stage 2 with or without pubic hair between 7 and 10 years; (2) a basal serum luteinizing hormone (LH) level ≧0.3 IU/L or peak LH after GnRH stimulation ≧5.0 IU/L, accepted as activation of the HPG axis; (3) advancement of bone age ≧1.5 years over chronological age; (4) recent increase in growth velocity (defined as >6 cm/year); (5) in the treated group, GnRHa (Leuplin Depot 3.75 mg S.C. injection) was administered every 28 days for at least 1 year.

Exclusion criteria included (1) girls born after 31 December 2005, excluding those who did not reach final height until 31 December 2020; (2) organic brain lesion confirmed by cranial magnetic resonance imaging; (3) underlying diseases that affect growth and puberty, such as Turner syndrome, congenital hypothyroidism, Down syndrome, and pseudohypoparathyroidism; (4) treatment with both GnRH analog and growth hormone; and (5) loss of critical data, such as height or bone age. Patients were further divided into treatment and control groups according to whether they received GnRHa.

### 2.2 Medical records

Participants were evaluated and followed up in outpatient clinics at 3-month intervals during treatment and follow-up. The chronological age (CA), height, weight, bone age (BA), and PAH of each patient were recorded at the beginning of the study and at follow-up appointments. Numbers 1 and 2 indicate the time points before and 1 year after treatment or follow-up, respectively. Body height was measured with a Harpenden Stadiometer to the nearest 0.1 cm. Bone age was checked and interpreted by experienced endocrinologists every 6 months using the standard of Greulich and Pyle Atlas (25). Tht was calculated as the average of the maternal and paternal height minus 6.5 cm. PAH was calculated using the Bayley and Pinneau tables for accelerated girls (26). Body mass index (BMI, kg/m^2^) was calculated as weight divided by height squared. Height SDS, weight SDS, BMI SDS, and PAH SDS were calculated according to chronological age. In addition, we tracked the FAH of patients using electronic records or telephone contacts. FAH was defined as a bone age >14 years or growth velocity less than 1 cm in the past year. Height gain (FAH-PAH1) and genetic height gain (FAH-Tht) were also calculated and compared between groups.

### 2.3 Data analysis

All data were evaluated using the Statistical Package for the Social Sciences (version 26.0; SPSS, Inc., Chicago, IL, USA) in the Biostatistics Department. Continuous variables were presented as median (range), and a nonparametric univariate analysis (Mann–Whitney U test) was applied to compare the differences in continuous parameters between the two groups. We analyzed ordinal repeated measures data, such as height and PAH at different time points by using a generalized estimating equation with Bonferroni all-pair multiple comparisons, and a *p*-value less than 0.05 was considered statistically significant. The Cochran–Mantel–Haenszel test was used in the analysis of categorical stratified data to evaluate the proportion of the population with a PAH1 initially exceeding the Tht, with a comparison of the proportion of the population with FAH reaching the Tht in both groups. Stepwise logistic regression models were used to identify significant independent contributing factors that predicted satisfactory adult height outcomes [height gain (FAH-PAH1) ≧ 5 cm]. Potential candidate factors were chosen according to literature review or univariate analysis. The area under the receiver operating characteristic curve (AUC) was used to evaluate the performance of each clinical parameter and the combination score. The cutoff value for the AUC was greater than 0.8.

### 2.4 Ethical consideration

The study was approved by the Mackay Children’s Hospital Research Ethics Committee (22MMHIS231e). We conducted a retrospective analysis using routine clinical and laboratory electronic data from Mackay Children’s Hospital, and the data were anonymously accessed. The IRB waived the need for informed consent forms.

## 3 Results

### 3.1 Growth parameters of the participants before and after one year of follow-up/treatment

A total of 204 patients were enrolled for analysis and divided into two groups based on whether they received treatment or not. The “treatment group” included 109 patients who received GnRH analog 3.75 mg (monthly for at least a year). In the treatment group, the median age at treatment was 8.7 (range 7.1–11.1) years, with a median duration of treatment of 2 (1.0–4.8) years. The median bone age at the end of treatment was 12.5 (9.5–14.0) years, with a PAH of 157.6 (147.3–170.4) cm. The median duration to menarche after cessation of GnRHa was 1 (0.1–3.9) year. The “control group” included 95 patients who did not receive any treatment but regularly visited outpatient clinics.

As shown in **Table 1**, the treatment group was significantly older at the time of inclusion (CA1 treatment vs. control, 8.7 vs. 8.4 years, *p* < 0.001). Height SDS, weight SDS, BMI SDS, and Tht were not significantly different between the groups. The treatment group had more advanced bone age (BA1-CA1, 2.7 vs. 2.2 years, *p* < 0.001), and shorter PAH1 (153.3 vs. 157.1 cm, *p* = 0.005), which was significantly lesser than the Tht (PAH1-Tht, −3.9 vs. −1.3 cm, *p* = 0.039). After 1 year of treatment and follow-up, the treatment group had slower growth velocity (4.6 vs. 7.3 cm, *p* < 0.001), more weight gain (BMI SDS2, 0.4 vs. 0.1, *p* = 0.002), slower bone age maturation (ΔBA/ΔCA, 0.5 vs. 1.5, *p* < 0.001), and improved PAH (PAH2-PAH1, 3.1 vs. −0.3 cm, *p* < 0.001).

**Table 1:** Comparison of the demographic characteristics and anthropometric data of the study groups.

	GnRHa *N* = 109 median	(min–max)	Control *N* = 95 median	(min–max)	*p*
CA1 (years)	8.7	(7.1–11.1)	8.4	(6.9–10.4)	**<0.001**
Height1 (cm)	137.0	(121.5–148.5)	133.2	(123.1–148.5)	**0.001**
HeightSDS1	1.1	(−1.9–3.3)	1.1	(−0.8–3.3)	0.548
Tht (cm)	157.0	(146.5–167.5)	157.0	(148.0–166.5)	0.630
Weight1 (kg)	33.5	(23.8–51.5)	30.0	(21.0–60.0)	**0.001**
WeightSDS1	0.6	(−0.8–3.6)	0.5	(−1.0–4.0)	0.619
BMI1 (kg/m^2^)	17.7	(14–25.1)	17.0	(12.9–29.0)	0.052
BMISDS1	0.2	(−1.3–3.6)	0.1	(−0.5–4.0)	0.331
BA1 (years)	11.5	(9–13.3)	10.8	(8.5–13.3)	**<0.001**
BA1-CA1 (years)	2.7	(0.9–4.5)	2.2	(1.0–4.1)	**<0.001**
PAH1 (cm)	153.8	(142.2–167.4)	157.1	(143.3–171.5)	**0.006**
PAH1-Tht (cm)	-3.9	(−15.3–11.1)	–1.3	(−13.1–15)	**0.039**
LHP (mIU/L)	15.6	(6–125.5)	11.5	(0.91–75.47)	**0.001**
CA2 (years)	9.7	(8.2–12.2)	9.3	(7.6–12.3)	**0.001**
Height2 (cm)	142.4	(126.8–152.8)	141.1	(128.0–158.5)	0.424
HeightSDS2	0.9	(−1.6–2.9)	1.1	(−0.8–3.4)	**0.017**
GV (cm/year)	4.6	(1.0–14.8)	7.3	(2.2–14.7)	**<0.001**
Weight2 (kg)	39.0	(24.6–57)	35.0	(25–78.8)	**<0.001**
WeightSDS2	0.7	(−1.3–3.6)	0.5	(−1.3–4.2)	0.231
BMI2 (kg/m^2^)	19.5	(13.8–27.3)	18.0	(13.6–31.9)	**<0.001**
BMI2SDS	0.4	(−1.4–3.4)	0.1	(−1.4–5.0)	**0.002**
BA2 (years)	12.0	(9.5–13.5)	12.0	(10.0–14.5)	0.490
BA2-CA2 (years)	2.3	(0.4–4.3)	2.6	(1.2–4.4)	**<0.001**
ΔBA/ΔCA*	0.5	(0–2.5)	1.5	(0–4.1)	**<0.001**
PAH2 (cm)	156.3	(145.7–170.0)	156.1	(139.3–168.9)	0.955
PAH2-PAH1 (cm)	3.0	(−8.1–9.0)	–0.3	(−11.2–12.7)	**<0.001**
PAH2-Tht (cm)	-1.7	(−11.4–15)	–2.2	(−11.2–13.64)	0.481
Age at menarche (year)	11.8	(8.6–14.4)	10.4	(8.1–15.7)	**<0.001**
At final adult height					
FAH (cm)	157.0	(144.6–169.0)	156.7	(139.0–168.0)	0.357
AHSDS	-0.3	(−2.6–1.7)	–0.5	(−3.7–1.5)	0.072
Weight (kg)	53.0	(38.0–90.0)	50.0	(37.0–90.0)	0.043
Weight SDS	-0.1	(−1.6–4.20)	–0.4	(−1.9–4.15)	**0.014**
BMI (kg/m^2^)	21.4	(16.5–33.2)	19.6	(14.5–35.3)	**<0.001**
BMISDS	0.1	(−1.3–3.8)	–0.4	(−2.0–4.4)	**<0.001**
FAH-PAH1 (cm)	4.0	(−8.8–12.6)	0.6	(−10.5–14.1)	**<0.001**
FAH-Tht (cm)	0.0	(−14.9–9.5)	–1.5	(−11.5–14.0)	**0.045**

Numbers 1 and 2 indicate the time points at:

1: early and fast puberty in the control group and before treatment in the treatment group

2: after 1-year follow-up in the control group and after 1 year of treatment in the treatment group

Values represent median (minimum–maximum).

GnRHa, gonadotropin-releasing hormone analog; CA, chronological age; Tht, target height; BA, bone age; BMI, body mass index; SDS, standard deviation score; PAH, predicted adult height; B, Tanner breast stage; LHP, LH peak level after GnRHa stimulation test; GV, growth velocity; FAH, final adult height.

*The bone maturation ratio (BMR) was calculated as the Δ bone age (BA2−BA1)/Δ chronological age (CA2−CA1) ratio.

The bold font in the table signifies that the p-value for this subgroup is less than 0.05.

The FAHs of the GnRHa and control groups were similar (157.0 vs. 156.7 cm, *p* = 0.357) and were not significantly different from Tht (FAH vs. Tht in the GnRHa group, 157.0 vs.157.0 cm; control group, 156.7 vs. 157 cm).

**Figure 1:**
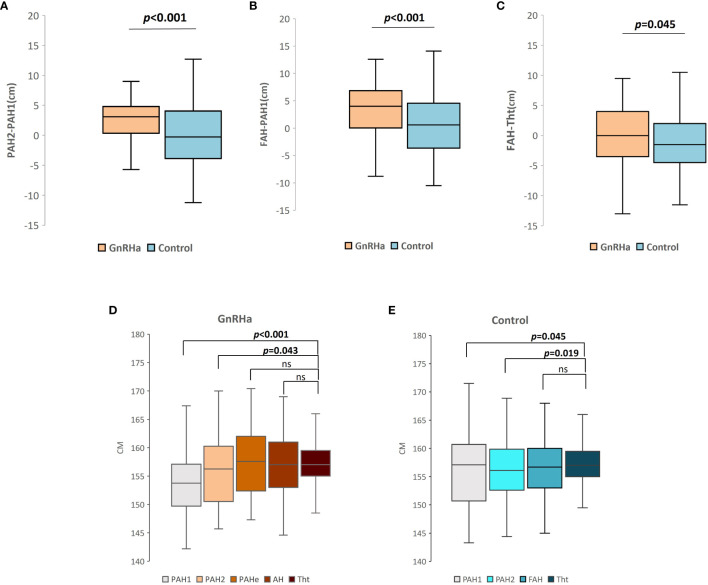
**(A)** Box plots of PAH2-PAH1 in two groups. **(B)** Box plots of height gain (FAH-PAH1) in two groups. **(C)** Box plots of genetic height gain (FAH-Tht) in two groups. **(D)** Box plot of PAH1, PAH2, PAHe*, FAH, and target height of treatment group. *PAHe, predicted adult height at the end of treatment. **(E)** Box plot of PAH1, PAH2, FAH, and target height of control group. ns, not significant.

Height gain (FAH-PAH1), genetic height gain (FAH-Tht), and the difference in PAH before and after follow-up/treatment (PAH2–PAH1) were significantly greater in the GnRHa group than in the control group (**Figures 1A–C**). Both the treatment and control groups eventually reached their Tht (**Figures 1D, E**).

**Figure 2:**
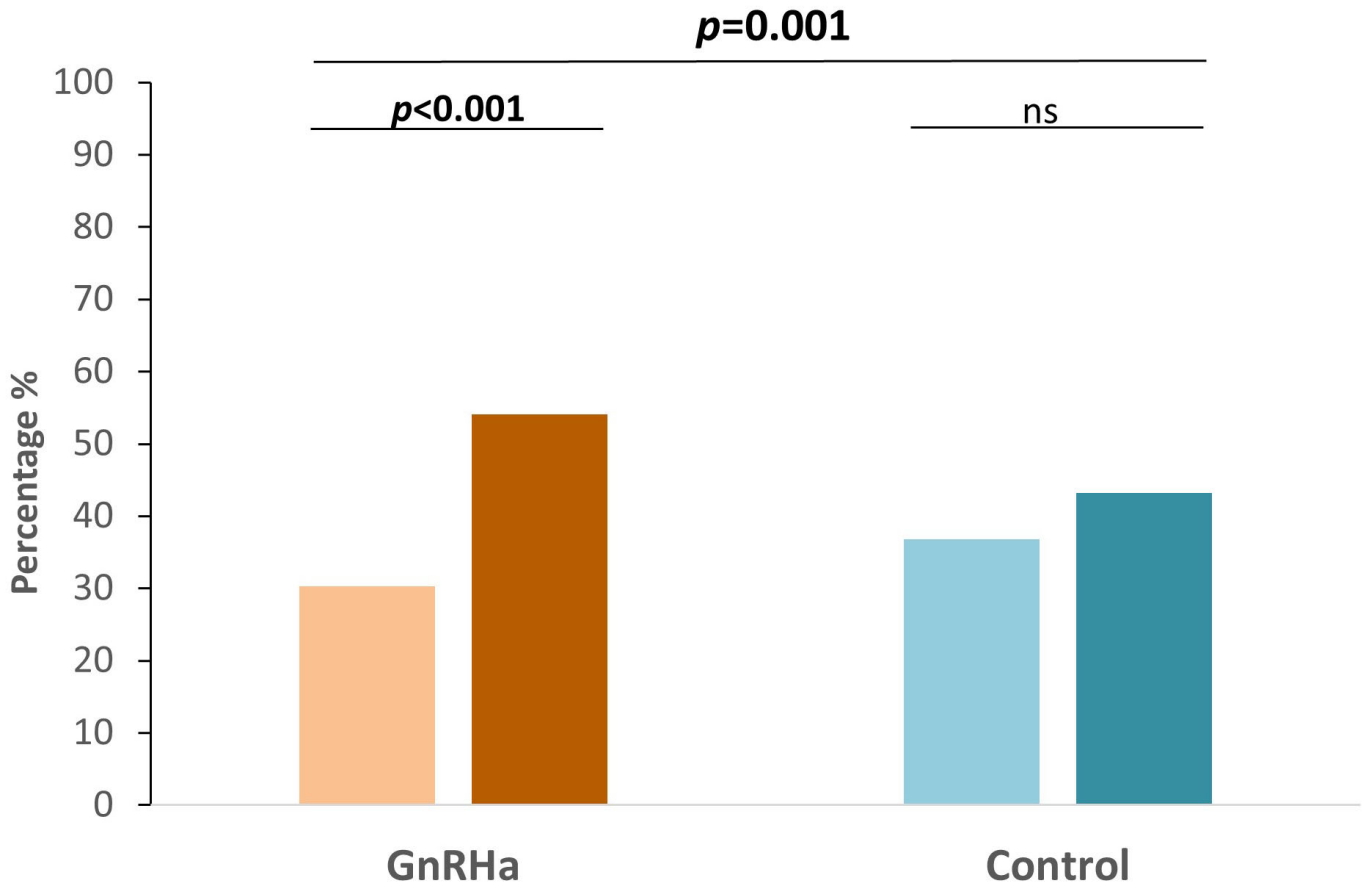
Bar plots of the percentage of patients predicted to reached Tht initially and the percentage of patients who eventually attained Tht in each group. ns, not significant.

In the subgroup analysis, FAH significantly improved after GnRHa treatment in those with PAH1 less than 153 cm and Tht (**Table 2**), and in those whose CA1 was between 8 and 9 years (**Table 3**). The same results were not observed in the younger group (CA1 less than 8 years), which might be attributed to small sample sizes. In addition, a higher proportion of subjects in the treatment group finally reached their Tht (FAH ≥ Tht) than the initial prediction (PAH1 ≥ Tht) (30.3% vs. 54.1%), while there were no differences in the control group (36.8% vs. 43.2%) (**Figure 2**).

**Table 2:** Subgroup analysis of FAH according to PAH1 and Tht.

	GnRHa n	FAH(cm) median	(min-max)	Control n	FAH(cm) median	(min-max)	*P*
PAH1≧Tht	33	160	(150.0-168.5)	35	158	(145.0-168.0)	0.125
PAH1<Tht and >155cm	16	160	(145.0-169.0)	23	159	(145.0-168.0)	0.563
PAH1<Tht and ≦155cm	60	155	(144.6-165.0)	37	154	(139.0-163.5)	0.051
	GnRHa n	FAH(cm) median	(min-max)	Control n	FAH(cm) median	(min-max)	*P*
PAH1≧Tht	33	160	(150.0-168.5)	35	158	(145.0-168.0)	0.125
PAH1<Tht and >153cm	27	160	(145.0-169.0)	26	159	(145.0-168.0)	0.943
**PAH1<Tht and≦153cm**	**49**	**154**	**(144.6-164.0)**	**34**	**152**	**(139.0-163.5)**	**0.041**

The bold font in the table signifies that the p-value for this subgroup is less than 0.05.

**Table 3:** Subgroup analysis of FAH according to CA1.

	GnRHa *n*	FAH (cm) median	(min–max)	Control *n*	FAH (cm) median		*p*
CA1 < 8 years	12	155	(144.6–166.0)	27	158	(148.0–168.0)	0.216
**8 ≦ CA1 < 9 years**	**65**	**158**	**(148.0–169.0)**	**57**	**155.4**	**(139.0–168.0)**	**0.004**
9 ≦ CA1 < 10 years	23	155	(145.0–165.0)	10	155.9	(148.0–162.0)	0.829
10 years ≦ CA1	9	154.5	(149.0–161.0)	1	156	(156.0–156.0)	0.861

The bold font in the table signifies that the p-value for this subgroup is less than 0.05.

### 3.2 Factors associated with a greater height gain

**Figure 3:**
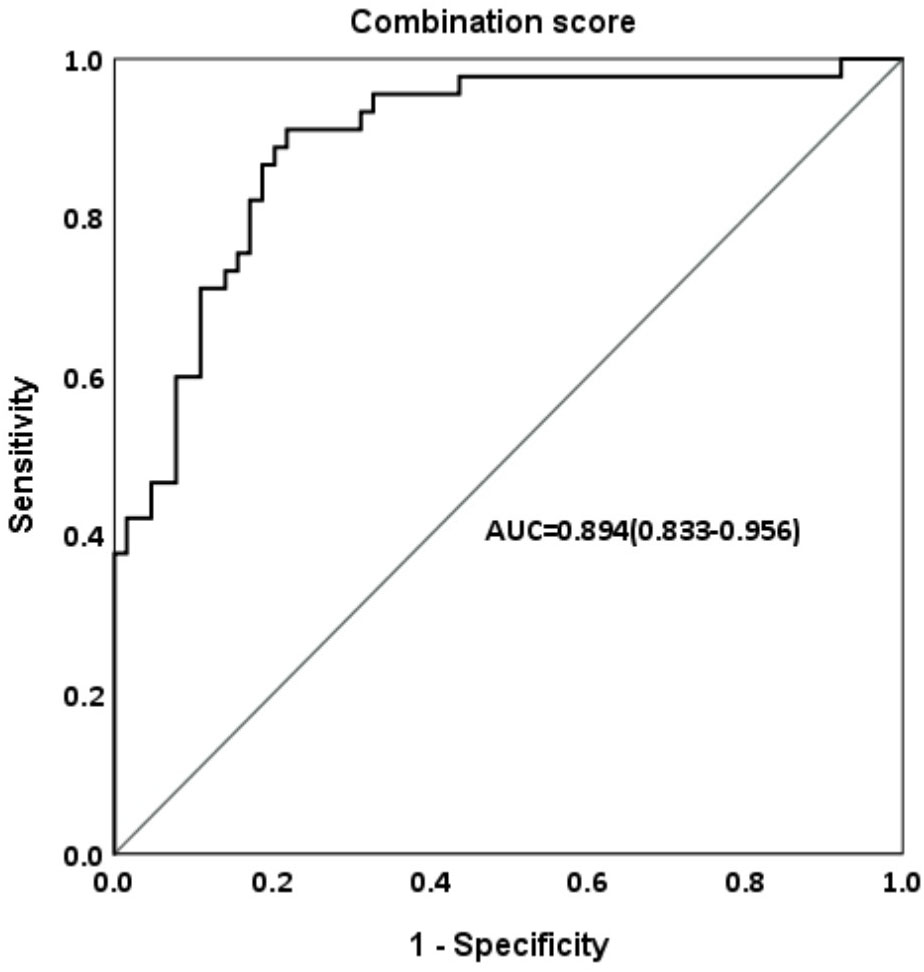
Area under the curve (AUC) of combination score in ROC analysis predicting FAH-PAH1≧5 cm in the treatment group.

When we defined satisfactory FAH outcome as FAH–PAH1 ≧5 cm, **Table 4A** shows that for all participants treated with the GnRH analog, HeightSDS1, Tht-PAH1, growth velocity (GV), and peak LH levels after the GnRH stimulation test are significantly associated with a greater height gain. In the treatment group, GV, Tht-PAH1, and CA1 were the significant factors. They were also qualified predictors by performing ROC curve analyses (AUC > 0.6) and the cutoff values were 4.55 cm/year, 4.95 cm, and 9.05 years, respectively, as determined by the maximum Youden indices (**Table 4B**). The prediction formula was then organized as follows: Prediction score = 5.087 + 0.639 * GV - 1.128 *CA1 + 0.323 * Tht-PAH1. The area under the curve was 0.894 (**Figure 3**).

**Table 4A:** Predictors of FAH-PAH1 ≧ 5 cm through multiple logistic regression Overall.

Variables	Adjusted OR	95% CI	*p*
Overall			
Untreated	1.00 (ref)		
Treated	5.32	1.96–14.45	**0.001**
HeightSDS1	2.11	1.81–3.73	**0.01**
Tht-PAH1 (cm)	1.48	1.31–1.68	**<0.001**
CA1 (years)	0.4	0.18–0.86	**0.018**
LHP (mIU/L)	0.94	0.90–0.98	**0.004**
Treatment group			
Tht-PAH1(cm)	1.38	1.21–1.58	**<0.001**
GV (cm/year)	1.9	1.23–2.94	**0.005**
CA1 (years)	0.32	0.14–0.75	**0.009**

The bold font in the table signifies that the p-value for this subgroup is less than 0.05.

**Table 4B:** Area under the curve AUC of variables in ROC analysis predicting FAH-PAH1 ≧ 5 cm in the treatment group.

Variables	AUC	95% CI	Cutoff values	Specificity (%)	Sensitivity (%)
Tht-PAH1 (cm)	0.803	0.723–0.884	>4.95	75.0	73.3
GV (cm/year)	0.72	0.623–0.818	>4.55	65.6	75.6
CA1 (years)	0.648	0.546–0.750	<9.05	42.2	88.9

## 4 Discussion

Our findings demonstrated that GnRHa did not result in a difference in the FAH compared to the control group in girls with early and fast puberty. However, GnRHa effectively restored the Tht in patients with an initially poor PAH. In addition, treatment with GnRHa also improved the final AH for those with (1) PAH1≦153 cm and less than the Tht and (2) girls aged 8–9 years at initial treatment. Furthermore, a significant proportion of patients who were originally considered to not exceed the Tht eventually reached their target height after GnRHa treatment. Finally, our results showed that poor initial PAH, rapid growth velocity during the first year, and younger age at treatment were important factors in attaining an FAH exceeding the initial PAH by 5 cm or more.

In our study, both groups reached their Tht, similar to previous studies with an untreated control group (4, 14, 15, 22, 27, 28). A meta-analysis evaluating the FAH also reported no significant differences between the groups (6, 23). However, another recent meta-analysis that included studies with larger sample sizes showed that GnRHa significantly increased FAH (7). Our study demonstrated that GnRHa increased the proportion of adults with a final height greater than Tht, whose initial PAH failed to reach the target height. After 1 year of treatment, slower bone age maturation and greater improvement in PAH eventually enabled them to attain the Tht. These results indicate that GnRHa was effective in restoring the height potential of those initially expected to have a significant height loss compared to their Tht.

In the subgroup analysis, treatment with GnRHa led to a significantly higher FAH in girls with a PAH less than Tht and less than 153 cm. A previous meta-analysis also performed a subgroup analysis with a PAH criterion of less than 155 cm and discovered no difference in adult height between the treatment and control group (6). We also performed a subgroup analysis with the criterion of <155 cm, which revealed results similar to those of the previous study. However, when we lowered the criterion to 153 cm, which was also compatible with our National Health Insurance payment standard (2), FAH was higher in the treatment group. We assumed that because a person’s target height represents the genetic potential to reach a certain height, those who were initially predicted to be equal to or higher than their Tht benefitted less from GnRHa, as a compensatory mechanism has been mentioned in previous studies; that is, the shorter height at the beginning of EP compared to the height at the beginning of older pubertal age was balanced by a greater gain in pubertal height, leading to an unimpaired adult height (14, 27). Our results again showed that GnRHa was most beneficial for early and fast pubertal girls with significant height loss, especially if they had a PAH of <153 cm.

Another subgroup analysis revealed better FAH in the treatment group when the chronological age was between 8 and 9 years, which accounted for the largest proportion of our study subjects. Most studies that recruited girls with EP emerging between 8 and 9 years did not support the benefits of GnRHa treatment. Ibanez et al. reported that most girls with normal birth weights managed to attain their Tht without treatment (16). However, there was no PAH in the article, indicating the potential presence of individuals within the study subjects who may not experience potential height loss. These individuals were not originally considered candidates for treatment. Lazar et al. with the control group concluded that GnRHa affects only the pace of puberty but not the final height (15). However, at initial treatment, the treatment group seemed to exhibit a greater potential height loss. Nevertheless, they still attained the target height after treatment, and their FAH was comparable to that of the control group. This finding aligns with our results.

In 2007, Lazar et al. discovered less post-treatment height gain when the onset of puberty was older than the initial puberty, that is, before 6 or 6–8 years (29). The age at initial treatment was almost 9 years in the EP group, which did not reach the Tht. Growth potential was greater in younger age groups because of the number of cell divisions until epiphyseal fusion and predetermined growth cessation, declining with age (30). Treatment initiation after the age of 9 years must be carefully evaluated because the treatment effect is difficult to predict. In our study, the same results were not observed in the younger groups, which could be due to the small sample size of this category.

Many studies have also focused on identifying factors that influence height outcomes after treatment. Factors considered to have a better influence on the prognosis of height include younger age at treatment initiation (29, 31, 32), better height SDS at the beginning (12, 13, 29) or end of treatment (12, 29, 33), better growth velocity during treatment (13), higher target height (29, 34), and longer treatment duration (13). The presence of GnRHa treatment was positively correlated with satisfactory height outcome, indicating that there was indeed a group of people who had a higher probability of obtaining a better height prognosis due to treatment. Another crucial factor was Tht-PAH1, which also demonstrated a positive correlation with satisfactory height outcome. This implies that patients with PAH with greater height disparity relative to Tht benefit more from GnRH treatment. Age at treatment initiation was inversely correlated with satisfactory height outcomes, implying that the younger the treatment, the more desirable the height gain. Growth velocity during the first year of treatment was a strong predictor; the faster the growth during treatment, the better the height prognosis. These variables help clinicians and parents determine whether girls with early and fast puberty should receive GnRHa treatment.

The strength of our study was that we enrolled a considerable number of untreated patients. This is the first study in Taiwan with a control group and a relatively large number of cases to discuss the effect of GnRHa on the FAH in girls with early and fast puberty. However, there are some limitations to our study. The retrospective design was its main focus. It was also a single-center study that raised a certain risk owing to selection bias for a relatively single and concentrated research population.

GnRHa is effective in restoring the Tht in some early and fast pubertal girls, especially those with poorly PAH (PAH lower than 153 cm and shorter than the target height). In addition, factors associated with the FAH exceeding the initial PAH by 5 cm or more included GnRHa therapy, PAH shorter than the Tht, age younger than 9 years, and faster growth velocity during the first year. In the case of girls experiencing EP and displaying a PAH close to their target height, healthcare providers should elucidate to both patients and parents that interventions provide little improvement to FAH. In most instances, regular monitoring would prove adequate.

## Data availability statement

The raw data supporting the conclusions of this article will be made available by the authors, without undue reservation.

## Ethics statement

The studies involving humans were approved by Mackay Children’s Hospital Research Ethics Committee. The studies were conducted in accordance with the local legislation and institutional requirements. The human samples used in this study were acquired from primarily isolated as part of your previous study for which ethical approval was obtained. Written informed consent for participation was not required from the participants or the participants’ legal guardians/next of kin in accordance with the national legislation and institutional requirements.

## Author contributions

C-HT: Formal Analysis, Software, Writing – original draft, Data curation, Writing – review & editing. W-HT: Writing – review & editing, Methodology, Supervision, Writing – original draft. Y-JL: Conceptualization, Project administration, Resources, Supervision, Writing – review & editing. C-YH: Conceptualization, Methodology, Resources, Supervision, Writing – review & editing. Y-LW: Resources, Supervision, Writing – review & editing. L-TW: Data curation, Investigation, Software, Writing – review & editing. C-HL: Data curation, Investigation, Writing – review & editing. B-WC: Investigation, Project administration, Resources, Writing – review & editing. F-SL: Conceptualization, Supervision, Writing – review & editing. Y-JC: Methodology, Software, Data curation, Writing – review & editing.
